# Antioxidant and Antiglycating Constituents from Leaves of *Ziziphus oxyphylla* and *Cedrela serrata*

**DOI:** 10.3390/antiox5010009

**Published:** 2016-03-17

**Authors:** Rizwan Ahmad, Niyaz Ahmad, Atta Abbas Naqvi, Vassiliki Exarchou, Atul Upadhyay, Emmy Tuenter, Kenn Foubert, Sandra Apers, Nina Hermans, Luc Pieters

**Affiliations:** 1 Natural Products and Alternative Medicines, College of Clinical Pharmacy, University of Dammam, P.O. Box 1982, Dammam 31451, Kingdom of Saudi Arabia; 2 Department of Pharmaceutics, College of Clinical Pharmacy, University of Dammam, P.O. Box 1982, Dammam 31451, Kingdom of Saudi Arabia; nanhussain@uod.edu.sa; 3 Department of Pharmacy Practice, College of Clinical Pharmacy, University of Dammam, P.O. Box 1982, Dammam 31451, Kingdom of Saudi Arabia; Atta_abbas@hotmail.com or Bg33bd@student.sunderland.ac.uk; 4 Natural Products & Food Research and Analysis (NatuRA), Department of Pharmaceutical Sciences, University of Antwerp, Universiteitsplein 1, 2610 Antwerp, Belgium; vasiliki.exarchou@ua.ac.be (V.E.); atul616@yahoo.com (A.U.); emmytuenter@hotmail.com (E.T.); kenn.foubert@uantwerpen.be (K.F.); sandra.apers@uantwerpen.be (S.A.); nina.hermans@uantwerpen.be (N.H.); luc.pieters@uantwerpen.be (L.P.)

**Keywords:** *Ziziphus oxyphylla*, Rhamnaceae, *Cedrela serrata*, Meliaceae, antioxidant, antiglycation, flavonoids

## Abstract

*Ziziphus oxyphylla* and *Cedrela Serrata* plants have a folkloric use in Pakistan for treatments of different ailments, *i.e.,* Jaundice, Hepatitis, Diabetes, and antimicrobial. Until now, none of the research studies have reported any phytochemical work on leaves of these two plants. This study aimed to isolate and perform phytochemical analysis in order to search for the constituent having the active role in treatment of the aforementioned ailments. A bioassay-guided fractionation and isolation procedure was used to isolate the concerned phytochemicals present in leaf extracts of *Z. oxyphylla* and *C. serrata*. The process involved the hyphenated techniques, *i.e.,* Flash Chromatography, Semi-Preparative HPLC/DAD, UPLC/MS, and NMR in order to isolate and elucidate the structure of the phytochemicals. Seven compounds (**1**–**7**) were isolated and identified as flavonoids, more in particular glycosides of quercetin and kaempferol. They showed DPPH scavenging activity, compound 3 (isoquercitrin) being the most active one with an IC50 of 10.8 µg/mL (positive control quercetin; IC50 3.6 µg/mL). The superoxide-radical scavenging and total antioxidant (ABTS) assays indicated IC50 values ranging from 200 to 910 µg/mL and 170 to 320 µg/mL, respectively (positive control quercetin: 374 and 180 µg/mL, respectively). Furthermore, these compounds had low IC50 values for inhibition of protein glycation (AGEs inhibition), ranging from 530 to 818 µg/mL, comparable to aminoguanidine (510 µg/mL) used as a positive control. This study resulted in the identification of seven flavonoid glycosides for the first time from the leaves of *Z. oxyphylla* and *C. serrata* with antioxidative and antiglycating activities.

## 1. Introduction

The diverse geographical and ecological environment of northern Pakistan allows the region to harbor a wide range of medicinal plants. These plants and their traditional usages are part of the natural and cultural heritage of Pakistan [1]. The trade of medicinal plants is increasing rapidly which is very important for the economy of the country. Nevertheless, a large part of this luxuriant flora has not been investigated in detail. This clearly indicates the need to unearth and systematically explore Pakistan flora. Two medicinal plants namely *Ziziphus oxyphylla* (Rhamnaceae) and *Cedrela serrata* (Meliaceae) which are commonly used to treat several diseases in Pakistan were selected for the present study.

*Ziziphus oxyphylla* is a small, almost glabrous tree or shrub [2] which is traditionally used in the treatment of inflammatory and painful complaints, microbial infections, allergy, diabetes, and as an antipyretic [3]. An ethno-ecological study of *Z. oxyphylla* indicated its anti-diabetic properties [4]. It has also been reported to possess anti-nociceptive and anti-pyretic activities in animal models [5] as well as anti-hypertensive properties [6]. Previous studies on the phytochemical contents of the plant have mainly focused on the isolation and identification of cyclopeptide alkaloids [7,8,9]. Antioxidant and antiglycation potential have been reported for *Z. oxyphylla* [10,11].

*Cedrela serrata* is a hardy tree, usually found in moist shady places at an altitude of 1000–2500 m. The leaves and bark of the plant are used to treat diabetes, fever, dysentery, as well as blood and skin diseases [12,13]. Recently, the antioxidant activity of a methanolic *C. serrata* leaf extract was determined *in vitro* in the DPPH free radical scavenging and DNA protection assays [14]. Antioxidant, antiglycating, and antimicrobial potential have also been reported [10,11]. However, there are no reports available on the characterization of its antioxidative constituents.

In the present study *Z. oxyphylla* and *C. serrata* leaf extracts were evaluated for their antioxidant activity. The most active extracts were explored for their antiglycation activity (inhibition of Advanced Glycation Endproducts—AGEs) taking into consideration the traditional use of both plants against diabetes. Indeed it has been well established that diabetic conditions are associated with increased oxidative stress and protein glycation [15,16,17]. Selected extracts were further fractionated in order to elucidate the structures of the individual constituents, and all isolated compounds were exploited for their antioxidant and antiglycation activities in order to provide insight into the active components of these plants.

## 2. Material and Methods

### 2.1. Chemicals and Reagents

NBT (Nitro blue tetrazolium chloride), DPPH (2,2-diphenyl-1-picrylhydrazyl radical), PMS (phenyl-methazonium-methosulphate), ABTS (2,2′-azino-bis(3-ethylbenzothiazoline-6-sulphonic acid), quercetin, reduced nicotinamide adenine dinucleotide (NADH) and potassium persulfate (K_2_S_2_O_8_) were purchased from Sigma Aldrich (St. Louis, MO, USA). Glucose, bovine serum albumin (BSA) and formic acid (FA) were obtained from Merck (Darmstadt, Germany). All solvents used were of High Performance Liquid Chromatography (HPLC) grade.

### 2.2. Plant Material

The leaves of *Z. oxyphylla* and *C. serrata* were collected from Swat Valley, northern Pakistan in 2009 and were identified by Mansoor Ahmad (Faculty of Pharmacy, University of Karachi, Karachi, Pakistan), where the voucher specimen (0012-2009/AZ; 0013-2009/BC) is deposited at the Herbarium (Centre for Plant Conservation), University of Karachi, Karachi, Pakistan. 

### 2.3. Extraction and Isolation

Leaf samples were dried and macerated three times in 80% methanol. Extracts with the same solvent were pooled together and concentrated under vacuum at 40 °C. Distilled water was added to the extract and the aqueous solution was partitioned successively with *n*-hexane, CHCl_3_, EtOAc, and *n*-butanol in order to prepare fractions with different polarities. The most active fractions, *i.e.,* the ethyl acetate fractions, selected on the basis of considerable antioxidant results, were subjected to further purification by flash chromatography (Reveleris X2 Flash system, Grace technologies, Ellicott City, MD, USA), ending up with sub-fractions of different weights. The gradient system used is shown in Table 1 as follows:

Four sub-fractions from the ethyl acetate fractions (E) of each plant were collected and dried under reduced pressure to yield 0.11 (ZE1), 0.14 (ZE2), 1.24 (ZE3) and 1.80 g (ZE4) for *Z. oxyphylla* (Z), and 0.14 (CE1), 0.38 (CE2), 1.92 (CE3), and 2.20 g (CE4) for *C. serrata* (C), respectively. All sub-fractions, obtained from flash chromatography, were again tested for their antioxidant activity in order to find the more active sub-fraction. ZE3, ZE4, and CE3, which were obtained in higher amounts, were found to be more active, and hence further subjected to semi-preparative HPLC-DAD (Agilent Technologies, Eindhoven, the Netherlands). The gradient development and isolation of compounds was accomplished, using a C18 column (250 × 10 mm internal diameter; 5 µm particle size; Phenomenex, Luna, Torrance, CA, USA) using 0.1% TFA in water (A) and MeOH (B); the flow rate was 3 mL/min, using the gradient in Table 2. Compounds **1**–**4** were isolated from sub-fractions ZE3 and ZE4, whereas compounds **5**–**7** were obtained from sub-fraction CE3. In addition, mixtures **M1** (48.1 mg) and **M2** (32.5 mg) were obtained from both ZE3 and ZE4 and the mixtures **M3** (20.6 mg) and **M4** (39.3 mg) were collected from CE3.

### 2.4. Biological Assays

#### 2.4.1. DPPH Scavenging Activity

DPPH scavenging activity of the fractions and isolated compounds was determined as reported previously [18] with slight modifications. Briefly, a 0.5 mM solution of DPPH in ethanol was prepared; 0.5 mL of fraction or test compound of different concentrations in EtOH (or EtOH itself as control) was added to 1.0 mL of the ethanolic DPPH· solution. The mixtures were shaken vigorously, left in the dark for 30 min, and the absorbance (at 517 nm) was measured. Scavenging activity could be calculated by the following equation:

%DPPH radical scavenging activity = (OD_C_ − OD_S_)/OD_C_ × 100

where OD_C_ and OD_S_ are the absorbance of control and test samples, respectively. The results were expressed as IC_50_ values and were calculated by linear regression analysis of tests conducted in triplicate. Quercetin was used as positive control.

#### 2.4.2. Superoxide-Radical Scavenging Activity by PMS-NADH System

The superoxide radical scavenging ability of isolated compounds and quercetin was assayed as previously described [19]. Different samples (0.1 mL) in MeOH were added to a mixture containing 0.5 mL NADH (105.6 µM) and 0.5 mL NBT (66 µM) in 0.1 M phosphate buffer (pH 7.4). The reaction was initiated by the addition of a volume of 0.5 mL of a solution of µM PMS, and after 5 min the absorbance was measured at 560 nm. The superoxide radical scavenging activity was measured as described above.

#### 2.4.3. Total Antioxidant Activity (ABTS^+^ Assay)

All extracts and isolated compounds were evaluated by the ABTS^+^ method [20]. The ABTS^+^ radical is generated by treating 7 mM ABTS with 2.45 mM K_2_S_2_O_8_ in the dark, at room temperature. The ABTS^+^ solution was first diluted with 80% EtOH to obtain an absorbance at 734 nm of 0.700 ± 0.050. A volume of 3.9 mL of this solution was then added to 0.1 mL of the test samples. After 6 min at room temperature the absorbance at 734 nm was recorded. Triplicate measurements were carried out, and the % decrease calculated as described above.

#### 2.4.4. Anti-Glycation Assay

The inhibition of protein glycation was measured as described [21], with some slight modifications. A volume of 200 µL each of the test compounds at different concentrations in DMSO was incubated with BSA (10 mg/mL) and glucose (500 mM) in 50 mM phosphate buffer (pH 7.4) at 60 °C for 48 h. The fluorescence (excitation 360 nm and emission 450 nm) due to the formation of AGEs was followed by spectrofluorometry (Tecan Infinite 200, Männedorf, Switzerland). In order to reduce the interference in the fluorescence signal by the compounds, parallel incubation at 4 °C was performed. The AGEs inhibition could be calculated as follows:

%AGEs inhibition = [1 − (S − Sb)/(C − Cb)] × 100

where S and C represent relative fluorescence units (RFU) for test samples (in DMSO) and control (test mixtures containing only DMSO) incubated at 60 °C, and S_b_ and C_b_ are RFU for samples incubated at 4 °C.

### 2.5. Statistical Analysis

One-way ANOVA was used to analyze the results. When differences were significant, means were separated by Tukey HSD range test at *p* = 0.01 with three replications. Statistical calculations were carried out in SPSS Statistics Version 20 (IBM Corporation, Marlborough, MA, USA).

## 3. Results

**Figure 1:**
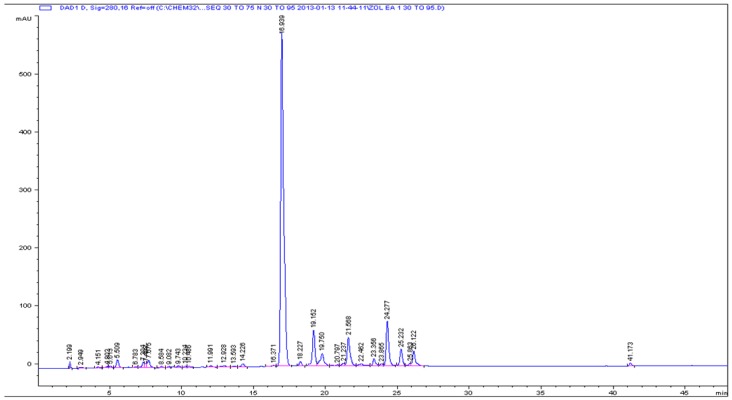
Analytical HPLC chromatogram for ZE3 sub-fraction.

**Figure 2:**
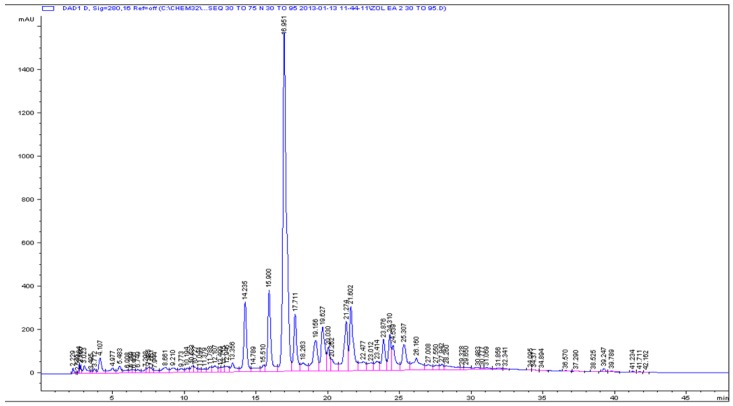
Analytical HPLC chromatogram for ZE4 sub-fraction.

**Figure 3:**
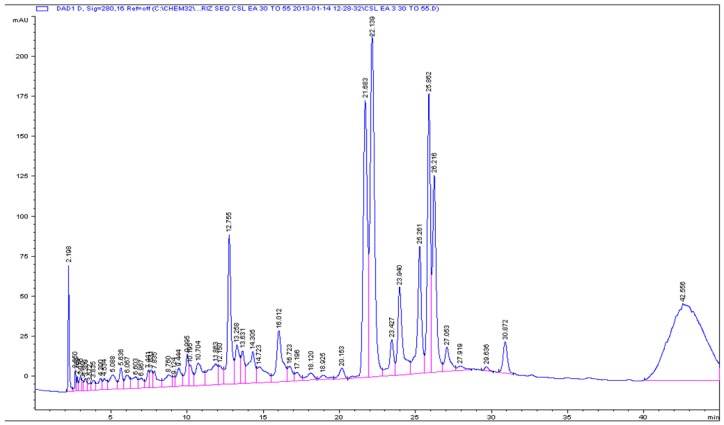
Analytical HPLC chromatogram for CE3 sub-fraction.

**Figure 4:**
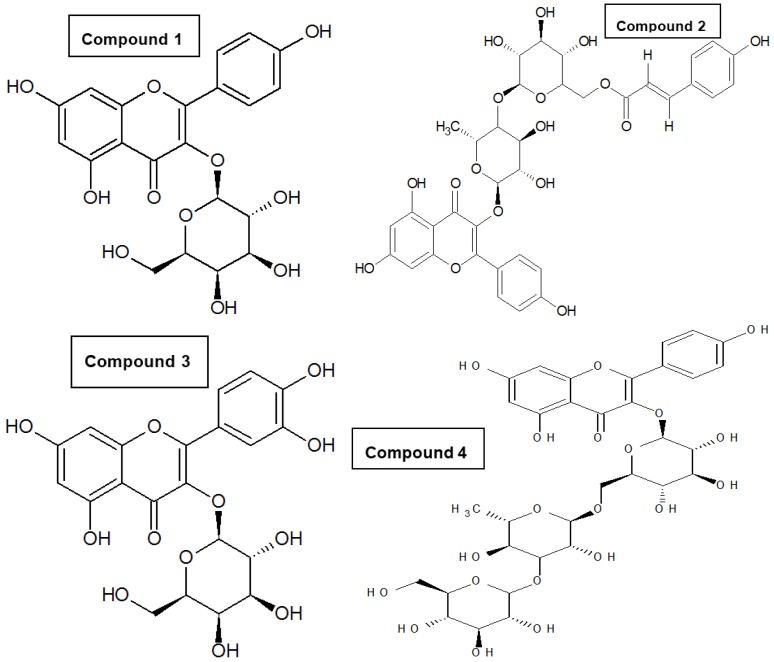

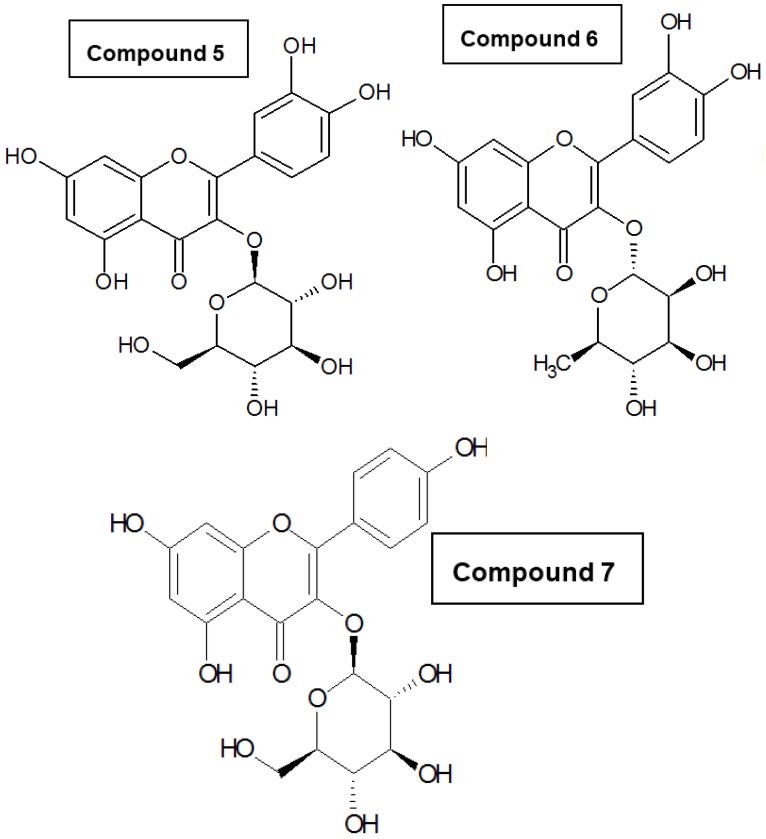
Structures for isolated compounds (**1–7**).

Our previous studies on crude extracts of *Z. oxyphylla* and *C. serrata* showed that their leaves are a very promising source of active components due to their antioxidant, antiglycation, and antimicrobial properties [10,11]. To further investigate the active components, the crude 80% methanolic leaf extract was partitioned successively with *n*-hexane, CHCl_3_, EtOAc, and *n*-butanol. All extracts were evaluated using DPPH assay. The ethyl acetate fractions of both plants showed high DPPH scavenging activity and accordingly low IC_50_ values of 3.0 ± 0.1 and 6.9 ± 0.8 µg/mL for *Z. oxyphylla* and *C. serrata*, respectively (Table 3). These fractions were also tested for their antiglycation activity. The results showed a significant activity with IC_50_ values of 0.60 ± 0.02 and 0.62 ± 0.02 mg/mL for *Z. oxyphylla* and *C. serrata*, respectively. Thus, the ethyl acetate fractions were selected for further purification by flash chromatography. Four sub-fractions ZE1–ZE4 and CE1–CE4 were obtained from each plant and their radical scavenging activity was checked. The DPPH assay indicated a high inhibitory activity for all sub-fractions (Table 4). In addition, their ^1^H-NMR spectra were obtained to provide a first insight of their phytochemical content. The spectra of the sub-fractions ZE3 and ZE4 of *Z. oxyphylla* and CE3 of *C. serrata* showed characteristic resonances in the aromatic region with patterns that could be attributed to flavonoids and therefore they were subjected to semi-preparative HPLC to provide the individual components. As a result, seven pure compounds were isolated and four were collected as mixtures; compounds **1**–**4** were isolated from ZE3 and ZE4, respectively. Similarly, mixtures **M1** and **M2** were isolated from the ZE3 sub-fraction. Compounds **5**–**7** were isolated from CE3 along with the mixtures **M3** and **M4**. The chromatograms developed are shown for ZE3 (Figure 1), ZE4 (Figure 2), and CE3 (Figure 3) respectively. The structures for the identified compounds are shown in Figure 4. 

Spectroscopic analysis of the isolated constituents led to their identification as flavonoid glycosides. Typically flavonoids show two characteristic UV bands at 260–270 nm and 340–350 nm. Flavonoids and their glycosides show typical ^1^H NMR aromatic spin–spin coupling patterns. The aglycones were identified as quercetin and kaempferol. Kaempferol shows the typical pattern of a para-disubstituted aromatic ring (AA’XX’), since there is no 3’-OH group, in contrast to quercetin (1,2,4-trisubstituted benzene pattern). The glycosidic moieties could be identified based on the chemical shifts and coupling constants of the H-1 protons.

Compound **1** showed two doublets (*J* = 8.7−8.9 Hz) corresponding to H-2’/H-6’ and H-3’/ H-5’ (ring B), and two doublets (*J* = 1.5−1.6 Hz) assigned to H-6 and H-8 (ring A), suggesting a kaempferol aglycone. Compared to kaempferol itself, H-6 and H-8 were slightly shifted, pointing towards a glycosyl residue in position 3. The anomeric signal at 5.14 ppm (d, *J* = 7.8 Hz) was in agreement with a β-configuration. The ^1^H NMR spectral data were in close agreement with those previously reported for kaempferol-3-*O*-β-galactoside (trifolin) [22,23,24].

In the same way compound **2** could also be identified as a 3-substituted kaempferol derivative. The most informative NMR signals for its structure elucidation were the signals at 7.63 and 6.38 ppm (doublets, *J* = 15.8 Hz), corresponding with H-7’’’ and H-8’’’ of a coumaroyl group, with a *trans* configuration as evident from this large coupling constant. The signals at 7.45 and 6.83 ppm were attributed to the protons of the coumaroyl aromatic ring. The signal at 5.23 ppm (doublet, *J* = 7.2 Hz) was attributed to H-1of a β-galactoside moiety, linked at C-3 of kaempferol. The signal of a singlet at 4.46 ppm was attributed to the rhamnoside moiety and the downfield shift of C-6 of galactoside (~5 ppm) indicated the (1→6) linkage between the two sugar units. The *trans-p-*coumaroyl substituent is connected to C-4 of the galactoside moiety as shown on the HMBC map. This compound was confirmed as kaempferol-3-*O*-rhamnosyl (1→6)-(4’’-*trans*-*p*-coumaroyl)-galactoside [25].

The ^1^H NMR spectrum of compound **3** revealed a quercetin-like B-ring. The anomeric proton of the sugar at 5.27 ppm and the presence of an anomeric carbon at 102.7 for C-1” in ^13^C-NMR (Table 5) confirmed this compound as quercetin-3-*O*-β-glucoside (isoquercetrin) [26,27].

Compound **4** showed the characteristic 1H-NMR signals of kaempferol and three anomeric protons resonating at 5.1 ppm (*d*, *J* = 7.3 Hz), 4.53 ppm (*d*, *J* = 1 Hz) and 4.35 ppm (d, *J* = 7.3 Hz) indicated a kaempferol triglycoside. This compound could be identified as kaempferol-3-*O*-glucosyl (1→2) rutinoside, reported before [28].

The ^1^H-NMR data of compound **5** were similar to those previously listed for quercetin-3-*O*-β-galactoside (hyperoside) [26,27].

Also compound **6** contained a quercetin moiety, as evident from the 1H-NMR spectral data. Moreover, the glycosidic part of the ^1^H NMR spectrum indicated the presence of rhamnose as deduced from the chemical shift of H-1 (5.33 ppm, small doublet, *J* = 2 Hz). The remaining glycosidic signals between 4.11 and 3.21 ppm and the doublet at 0.92 ppm (methyl group of rhamnose) supported the presence of quercitrin (quercetin-3-*O*-α-rhamnoside) [29,30].

Compound **7** also showed the characteristic ^1^H-NMR AA’XX’ pattern of a kaempferol moiety. The presence of a doublet at 5.26 ppm (*J* = 7.3 Hz), assigned to the anomeric proton, directly correlated with a carbon at 100.8 ppm (Table 5) confirmed the structure of kaempferol-3-*O*-β-glucoside (astragalin).

Mixtures **M1**, **M2,** and **M3** consisted of two compounds (quercetin-3-*O*-glucoside and qurecetin-3-*O*-galactoside) in different ratios, whereas mixture **M4** contained quercetin-3-*O*-rhamnoside and kaempferol-3-*O*-galactoside in a ratio of almost 1:1.

All compounds were evaluated in the DPPH, PMS superoxide and ABTS^+^ assays (Table 6). Compound **3** exhibited the highest DPPH scavenging activity (IC_50_: 10.8 ± 0.7 µg/mL). Interestingly, the mixtures **M1** (5.3 ± 0.3 µg/mL), and **M3** (4.4 ± 0.3 µg/mL) had better scavenging activity than the isolated compounds and were similar to quercetin (3.6 ± 0.7 µg/mL) (*p* = 0.05; Table 6). Similarly, the IC_50_ values for PMS superoxide radical scavenging activity of isolated compounds ranged from 381–910 µg/mL. Compound **6** (IC_50_ 381 ± 6) exhibited comparable superoxide scavenging activity to quercetin (IC_50_ 374 ± 6 µg/mL; *p* = 0.05; Table 6) whereas the mixtures **M1** and **M3** exhibited significantly better scavenging activity (IC_50_ 207 ± 3 and 200 ± 3 µg/mL, respectively), than the positive control quercetin (*p* = 0.05; Table 6). The total antioxidant activity measured as ABTS radical scavenging ability showed that compounds **3**, **5,** and **6** and all the mixtures (**M1**–**M4**) had similar results to quercetin (180 ± 5 mg/mL) (*p* = 0.05; Table 6).

The ability of isolated compounds to inhibit protein glycation was evaluated using the BSA-glucose assay. In this assay BSA is considered as a model protein, whereas glucose is representative for all glycation agents. Compound **2** showed the highest inhibitory activity with an IC_50_ value of 530 ± 19 µg/mL (Table 6). On statistical analysis, compounds **1**–**3**, **5**, **6** and mixture **M3** had similar activities when compared with the positive control aminoguanidine (IC_50_ 510 ± 18 µg/mL; *p* = 0.05; Table 6).

### Identification of Isolated Compounds

^1^H, ^13^C-NMR and two-dimensional NMR (COSY, HSQC, and HMBC) experiments of the isolated compounds were recorded on a Bruker DRX 400 instrument equipped with a z-gradient 5 mm dual probe using standard Bruker pulse sequences (Rheinstetten, Germany). All samples were dissolved in deuterated methanol (CD_3_OD, 99.5% D).

ESI-MS spectra were obtained on a triple quad UPLC-MS system (Waters, Milford, MA, USA) with an ESI source. Direct infusion of the samples was performed. The optimized conditions were: capillary (4000 V), extractor (4 V), cone (90 V) voltage, source (80 °C), desolvation temperature (450 °C), RF lens 0.1 V, gas flow rate for desolvation (1000 L/h) and cone (50 L/h). The data was processed using Masslynx software (version 4.1) (available in the UPLC-MS system).

*Kaempferol-3-O-β-galactoside (trifolin) (**1**) (19.7 mg).* ESI-MS 447 [M − H]^−^. ^1^H NMR, δ ppm: 8.08 (2H, d, *J* = 8.9 Hz, H-2’and H-6’), 6.87 (2H, d, *J* = 8.7 Hz, H-3’and H-5’), 6.44 (1H, d, *J* = 1.6 Hz, H-8), 6.2 (1H, d, *J* = 1.5 Hz, H-6), 5.14 (1H, d, *J* = 7.8 Hz, H-1”), 3.2–3.9 (6H, m, H-2”, H-3”, H-4”, H-5”and H-6”).

*Kaempferol-3-O-rhamnosyl (1→6) (4’’-trans-p-coumaroyl) galactoside (**2**) (7.8 mg).* ESI – MS: 739 [M − H]^−^. ^1^H NMR δ ppm: 8.10 (2H, d, *J* = 8.7 Hz, H-2’, H-6’), 7.64 (1H, d, *J* = 15.8 Hz, H-7’’’coum), 7.48 (2H, d, *J* = 8.5 Hz, H-2’’’, H-6’’’coum), 6.91 (2H, d, *J* = 8.7 Hz, H-3’, H-5’), 6.83 (2H, d, *J* = 8.5 Hz, H-3’’’, H-5’’’coum), 6.41 (1H, d, *J* = 1.6 Hz, H-8), 6.38 (1H, d, *J* = 15.8 Hz, H-8’’’coum), 6.21 (1H, d, *J* = 1.5 Hz, H-6), 5.35 (1H, s, H-4 gal), 5.23 (1H, d, *J* = 7.2 Hz, H-1 gal), 4.46 (1H, s, H-1 rha), 3.9–3.2 (sugar protons), 1.05 (3H, d, *J* = 6.2 Hz, H-6 rha).

*Quercetin-3-O-β-glucoside (**3**) (25.3 mg).* ESI–MS: 463 [M − H]^−^, ^1^H NMR δ ppm: 7.70 (1H, d, *J* = 2 Hz, H-2’), 7.57 (1H, dd, *J*_1_ = 8.4 Hz, *J*_2_ = 2.1 Hz, H-6’), 6.85 (1H, d, *J* = 8.6 Hz, H-5’), 6.39 (1H, d, *J* = 1.9 Hz, H-8),6.19 (1H, d, *J* = 1.9 Hz, H-6), 5.27 (1H, d, 7.82 Hz, H-1”), 3.49–3.65 (6H, m, H2”–H6”).

*Kaempferol-3-O-glucosyl (1→2) rutinoside (**4**) (35.7 mg).* ESI – MS: 755 [M − H]^−^. ^1^H NMR δ ppm: 8.10 (2H, d, *J* =8.8 Hz, H-2’and H-6’), 6.88 (2H, d, *J* = 8.8 Hz, H-3’and H-5’), 6.44 (1H, d, *J* = 1.6Hz, H-8), 6.2 (1H, d, *J* = 1.5 Hz, H-6), 5.01 (1H, d, *J* = 7.8 Hz, H-1 glc), 3.2–3.9 (5H, m, H-2 – H-6 glc), 4.53 (1H, H-1 rha), 3.2–3.9 (5H, m, H2-H6 rha), 4.35 (1H, d, *J* = 7.8 Hz, H-1 glc), 3.2–3.9 (5H, m, H2 – H6 glc), 0.95 (3H, d, *J* = 6.2 Hz, H-6 rha).

*Quercetin-3-O-β-galactoside (hyperoside) (**5**) (28.4 mg).* ESI – MS: 463 [M − H]^−^. ^1^H NMR δ ppm: 7.84 (2H, m, *J* = 2 Hz, H-2’), 7.57 (2H, dd, *J* = 8.4 and 2.1 Hz, H-6’), 6.85 (1H, d, *J* = 8.6 Hz, H-5’), 6.39 (1H, d, *J* = 1.9 Hz, H-8), 6.19 (1H, d, *J* = 1.9 Hz, H-6), 5.17 (1H, d, *J* = 7.8 Hz, H-1”), 3.65 (2H, br d, *J* = 11.9 Hz, H-6”), 3.49–3.83 (4H, m, H-2”, H-3”, H-4” and H-5”).

*Quercitrin (quercetin-3-O-α-rhamnoside) (**6**) (33.5 mg).* ESI – MS: 447 [M − H]^−^. ^1^H NMR δ ppm: 7.32 (1H, d, *J* = 2Hz, H-2’), 7.30 (1H, dd, *J* = 2.1 Hz, *J* = 8.4 Hz, H-6’), 6.90 (1H, d, *J* = 8.6 Hz, H-5’), 6.36 (1H, d, *J* = 1.9 Hz, H-8), 6.19 (1H, d, 1.9 Hz, H-6), 5.33 (1H, d, *J* = 2 Hz, H-1”), 4.11–3.21 (4H, m, H-2’’, H-3’’, H-4’’, H-5’’), 0.92 (3H, d, *J* = 6 Hz, H-6”).

*Kaempferol-3-O-β-glucoside (astragalin) (**7**) (12.6 mg).* Yellow powder: ESI – MS: 447 [M − H]^−^. ^1^H NMR) δ ppm: 8.05 (2H, d, *J* = 8.9 Hz, H-2’ and H-6’), 6.87 (2H, d, *J* = 9 Hz, H-3’and H-5’), 6.4 (1H, d, *J* = 2.0 Hz, H-8), 6.2 (1H, d, *J* = 1.7 Hz, H-6), 5.26 (1H, d, *J* = 7.3 Hz, H-1”), 3.2–3.9 (5H, m, H-2”- H6”).

## 4. Discussion

The isolated compounds exhibited different antioxidant activities due to the number of hydroxyl groups present in the aromatic ring. Qurecetin-3-*O*-galacatoside had better antioxidant activity than quercetin-3-*O*-glucoside. These results show that quercetin glycosides have better antioxidative properties than kaempferol glycosides, which may be due to the presence of a higher number of hydroxyl groups in the aromatic ring. Interestingly, the mixtures had significantly better activity than the isolated pure compounds. This may be due to the synergistic effects of the flavonoid glycosides.

Advanced glycation endproducts are involved in many diseases such as diabetes-related complications, atherosclerosis, neurodegenerative diseases (Alzheimer’s disease), and physiological ageing. Since hyperglycation is considered to increase oxidative stress, glycation and oxidation appear to be inextricably linked [31]. Here we therefore investigated the anti-glycation efficacy of the antioxidant compounds. Most of the isolated compounds had similar activities compared to aminoguanidine, and therefore these compounds may have further uses in drugs designed as AGE inhibitors. Unlike in the antioxidative assays, the mixtures did not exhibit better antiglycating activity than the pure compounds, indicating that there is no significant role of synergism in the antiglycation activity of these compounds. Previous study has shown that the cyclopeptide alkaloids from roots and stem of *Z. oxyphylla* have anti-glycation properties [8]. Here we describe flavonoid glycosides as AGEs inhibitors from the leaves of this plant.

## 5. Conclusions

In summary, seven pure compounds and four mixtures of flavonoid glycosides with antioxidant properties have been reported for the first time from the leaves of *Z. oxyphylla* and *C. serrata*, of which the mixtures **M1**, **M2,** and **M3** had superior activity to quercetin in the antioxidant assays. The anti-glycation properties of these compounds indicated that they may have possible applications in the prevention of diabetic complications related to excessive glycation reactions. Our results may be useful at least in part for the pharmacological explanation of the use of *Z. oxyphylla* and *C. serrata* in folk medicine in Pakistan.

## Figures and Tables

**Table 1:** Flash chromatography gradient system used.

Time (min)	Solvents	%2nd Solvent
0	AB	0
45	AB	100
0.5	BC	0
30	BC	50

**Table 2:** Gradient system of isolated compounds from different sub-fractions.

ZE3		ZE4		CE3	
Time	%B	Time	%B	Time	%B
0	30	0	30	0	30
40	50	45	50	10	40
44	100	49	100	20	40
50	100	54	100	28	45
52	30	56	30	30	100
57	30	60	30	35	100
				37	30
				42	30

Wave length = 280 nm.

**Table 3:** DPPH radical scavenging activity (IC_50_, µg/mL) of different fractions of *Z. oxyphylla* and *C. serrata.*

	*n*-Hexane	Chloroform	Ethyl acetate	*n*-Butanol	Aq. MeOH
*Z. oxyphylla*	85.0 ± 1.2	4740 ± 24	3.0 ± 0.1	42.1 ± 0.4	64.2 ± 1.1
*C. serrata*	899.7 ± 9.4	473.0 ± 2.4	6.9 ± 0.8	510.5 ± 23.6	353.4 ± 12.1

**Table 4:** DPPH radical scavenging activity (IC_50_, mg/mL) of sub-fractions of the ethyl-acetate fraction of *Z. oxyphylla* and *C. serrata*.

	E1	E2	E3	E4
*Z. oxyphylla*	1.43 ± 0.10	0.46 ± 0.09	0.08 ± 0.01	0.43 ± 0.08
*C. serrata*	0.09 ± 0.01	0.08 ± 0.01	0.13 ± 0.03	0.14 ± 0.02

**Table 5:** ^13^C NMR spectral data of isolated compounds (**1**–**3**, **5**–**7**) in CD_3_OD (δ_C_, ppm, 400 MHz).

	1	2	3	5	6	7
2	157.1	158.1	157	157	158.0	156.42
3	134.2	133.8	135	135	135.8	133.20
4	178.3	178	178.7	178.7	179.65	177.53
5	161.7	161.8	161	161	163.2	161.24
6	98.5	98.5	98	98	99.50	98.85
7	164.6	164.8	164.4	164.4	164.70	164.33
8	93.3	93.4	94	94	94.5	93.76
9	157.6	157.7	157	157	158.40	156.42
10	104.2	104.1	104	104	105.8	104.01
1'	121.3	121.4	-	-	122.6	120.89
2'	131.0	130.9	116.3	116.3	116.1	130.96
3'	114.7	114.6	144	144	145.8	115.21
4'	160.2	160.1	148.6	148.6	149.0	160.10
5'	114.7	114.6	114.6	114.6	116.7	115.21
6'	131.0	130.9	121.4	121.4	122.9	130.96
Glu-1''	-		102.7	103.8	-	100.87
2''	-		71.6	71.6	-	74.24
3''	-		75.6	75.6	-	77.58
4''	-		68.4	68.4	-	69.89
5''	-		73.5	73.5	-	76.46
6''	-		60.4	60.4	-	60.83
Gal-1''	103.5	103.3	-	-	-	-
2''	71.6	71.8	-	-	-	-
3''	73.6	72.0-	-	-	-	-
4''	68.6	69.8	-	-	-	-
5''	75.7	72.5	-	-	-	-
6''	60.6	65.5	-	-	-	-
Rha-1''	-	100.5	-	-	102.7	-
2''	-	70.6	-	-	71.5	-
3''	-	70.6	-	-	72.1	-
4''	-	72.2	-	-	73.0	-
5''	-	68.4	-	-	71.3	-
6''	-	17.0	-	-	17.8	-
O-coumaric	-	-	-	-	-	-
1''	-	125.2	-	-	-	-
2''	-	129.9	-	-	-	-
3''	-	115.3	-	-	-	-
4''	-	159.8	-	-	-	-
5''	-	115.3	-	-	-	-
6''	-	129.9	-	-	-	-
7''	-	145.7	-	-	-	-
8''	-	113.4	-	-	-	-
9''	-	167	-	-	-	-

The symbols (') denotes the doublet found at Ring-B, *i.e*., Prime position whereas ('') shows the *trans* positions at anomeric carbons.

**Table 6:** DPPH, superoxide, total antioxidant activity (ABTS) scavenging, and advanced glycation endproducts (AGEs) inhibitory activity of compounds **1**–**7** (IC_50_ values).

	DPPH	Superoxide	ABTS	AGEs
	(IC_50_, µg/mL)	(IC_50_, µg/mL)	(IC_50_, µg/mL)	(IC_50_, µg/mL)
**1**	17.8 ± 1.1 ^d,^*	910 ± 14 ^h^	230 ± 7 ^c^	559 ± 20 ^a,b,c^
**2**	30.5 ± 1.9 ^e^	512 ± 8 ^f^	320 ± 9 ^d^	530 ± 19 ^a,b^
**3**	10.8 ± 0.7 ^c^	400 ± 6 ^c^	170 ± 5 ^a^	556 ± 19 ^a,b,c^
**4**	19.8 ± 1.2 ^d^	410 ± 6 ^c^	200 ± 6 ^b^	574 ± 20 ^b,c^
**5**	18.9 ± 1.1 ^d^	431 ± 7 ^d^	180 ± 5 ^a^	548 ± 19 ^a,b,c^
**6**	19.2 ± 1.2 ^d^	381 ± 6 ^b^	170 ± 5 ^a^	554 ± 19 ^a,b,c^
**7**	40.4 ± 2.5 ^f^	482 ± 7 ^e^	240 ± 7 ^c^	818 ± 29 ^d^
**M1**	5.3 ± 0.3 ^a,b^	207 ± 3 ^a^	170 ± 5 ^a^	586 ± 21 ^b,^^c^
**M2**	8.0 ± 0.5 ^b^	713 ± 11 ^g^	170 ± 5 ^a^	589 ± 21 ^c^
**M3**	4.4 ± 0.3 ^a^	200 ± 3 ^a^	170 ± 5 ^a^	541 ± 19 ^a, b, c^
**M4**	13.4 ± 0.8 ^c^	410 ± 6 ^c^	170 ± 5 ^a^	593 ± 21 ^c^
Quercetin	3.6 ± 0.6 ^a^	374 ± 6 ^b^	180 ± 5 ^a^	not tested
Aminoguanidine	not tested	not tested	not tested	510 ± 18 ^a^

* Different superscript letters in the same column indicate a significant difference (Tukey’s test; *p* = 0.05). The letters ^a–h^ denotes the comparative IC_50_ value of different isolated compounds with quercetin (standard drug), *i.e*., lower IC_50_ value (more activity) to higher IC_50_ value (less activity).
